# Modeling TCR‐Epitope Recognition Specificity: What We Should Learn to Succeed

**DOI:** 10.1111/imr.70140

**Published:** 2026-07-09

**Authors:** David Gfeller, Julien Racle, Rita Ann Roessner

**Affiliations:** ^1^ Department of Fundamental Oncology University of Lausanne Lausanne Switzerland; ^2^ Swiss Institute of Bioinformatics (SIB) Lausanne Switzerland; ^3^ Agora Cancer Research Centre Lausanne Switzerland

**Keywords:** computational immunology, machine learning, T‐cell epitope recognition, T‐cell receptor

## Abstract

T‐cell recognition of infected or malignant cells is central to both spontaneous and therapy‐induced cellular immune responses against pathogens and cancer. This recognition is elicited by the interaction between T‐Cell Receptors (TCRs) and epitopes, which consist of antigenic peptides displayed on major histocompatibility complex molecules. TCR‐epitope interactions are characterized by high diversity in TCR and epitope sequences and high structural flexibility in TCR loops. As a result, deciphering the rules of TCR‐epitope recognition specificity and accurately predicting these interactions remains challenging. Here, we review the different strategies developed to predict TCR‐epitope recognition, classify the principal computational frameworks, examine the data modalities on which they depend and discuss their current limitations. We then synthesize key conceptual insights that have emerged from recent research and outline how these lessons should inform the design of future experiments and next‐generation computational tools.

## 1 Introduction

### 1.1 T‐Cell Epitope Recognition

T‐cell epitope recognition is elicited by the interaction between T‐cell Receptors (TCRs) expressed in T cells and antigenic peptides displayed on Major Histocompatibility Complex (MHC) molecules on the surface of target cells, like infected cells, cancer cells, or antigen presenting cells. This interaction triggers a series of molecular events that can lead to the killing of the target cells by cytotoxic CD8 T cells, the activation of CD8 T cells by specific CD4 T cells, or the regulation of immune responses by CD4 regulatory T cells. These mechanisms play a central role in mediating and modulating cellular immune responses, as demonstrated by the clinical success in cancer immunotherapy of antibodies blocking immune inhibitory receptors [1, 2, 3], of vaccines eliciting strong T‐cell immunity [4, 5, 6, 7] and of adoptive transfer of cancer‐specific T cells [8, 9, 10].

### 1.2 TCR‐Epitope 3D Structures

TCRs are heterodimeric molecules with one alpha and one beta chain in most T cells, or one gamma and one delta chain in γδT cells. TCRs bind epitopes, which consist of peptides displayed on MHC molecules, through a complex interface. Crystallographic studies have demonstrated a key role for several loops in TCR alpha and beta chains [11, 12]. These loops are referred to as Complementarity Determining Regions (CDR) 1, 2, and 3. Residues in these loops mediate the majority of interactions with the epitopes, although evidence exists that residues outside of these loops can in some cases directly contact the epitopes [13, 14]. On average, the same number of residues in contact with the epitope is found in the alpha and beta chain [15]. All CDR loops are characterized by a high structural flexibility, and their precise binding modes differ substantially across TCRs recognizing different epitopes. This high flexibility, together with the diversity in amino acid composition and length of these loops, has represented an important challenge for predicting TCR‐epitope 3D structures. However, the development of deep learning–based protein structure and interaction prediction frameworks, such as AlphaFold3 (AF3) [16], is having a profound impact in this field [17, 18, 19].

### 1.3 TCR Sequence Diversity

**FIGURE 1:**
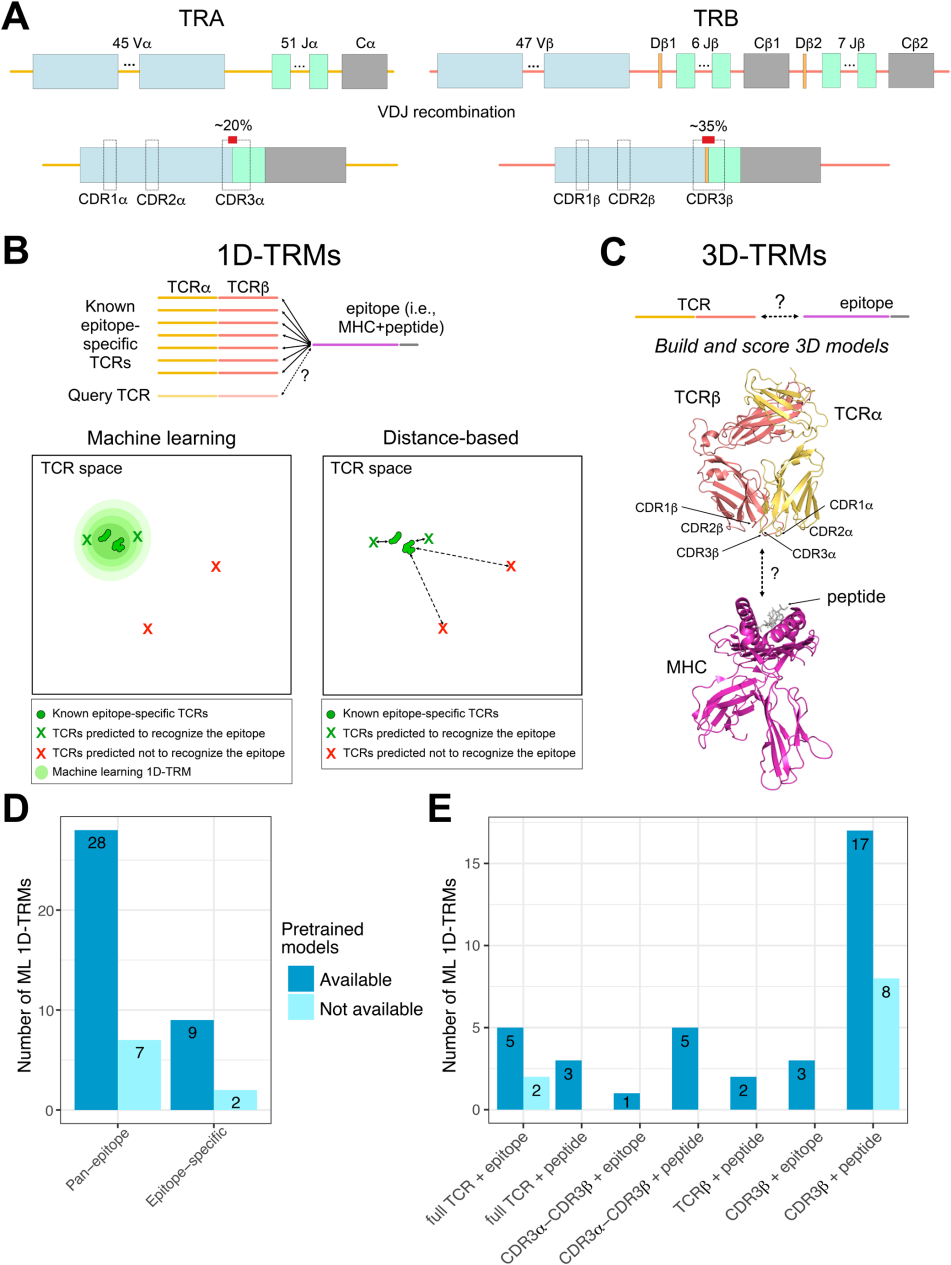
(A) Representation of the V(D)J recombination process, together with the constant (C) regions, on both chains. Numbers indicate the number of functional V and J genes. CDR loops are shown with dashed rectangles. The red rectangles at the V(D)J junctions within the CDR3 loops illustrate the average fraction of CDR3 residues that are not determined by V and J gene choices. (B) Illustration of 1D‐TRMs, including those that rely on a machine learning model (illustrated with the green shaded ellipses for an epitope‐specific tool) and those that use TCR distance metrics (illustrated with the arrows). (C) Illustration of 3D‐TRMs that rely on predicted 3D models of TCR‐epitope complexes for predicting TCR‐epitope recognition. (D) Number of machine learning (ML) 1D‐TRMs with pan‐epitope or epitope‐specific architectures and providing or not pretrained models. (E) Number of machine learning 1D‐TRMs as a function of the different input types for the TCR and epitope sequences. Cases using V + CDR3 + J or CDR1 + CDR2 + CDR3 for both chains have been merged under the “full TCR” label.

TCRs exhibit a very high sequence diversity, with estimates ranging from 10^16^ to 10^60^ (ref. [20, 21, 22, 23]). This diversity arises from a process called V(D)J recombination. First, a specific V and a specific J gene, plus a short D segment for the beta chain, are selected from the set of V and J genes encoded at the TCR loci (TRA for alpha chain and TRB for beta chain) and joined together with the constant (C) region for each chain (Figure 1A). Amino acid sequences for the different V and J genes are available in databases such as the ImMunoGeneTics (IMGT) database [24]. The number of possible Vα‐Jα‐Vβ‐Jβ combinations is in the order of 10^6^. Additional diversity occurs at the V(D)J junction, a region located within the CDR3 loops, where nucleotide insertions and deletions can give rise to variable amino acid sequences beyond those resulting from different choices of V and J genes (Figure 1A). The very large diversity of TCR sequences enables different T cells to recognize many different epitopes.

CDR1 and CDR2 residues, as well as 80% of CDR3α and 65% of CDR3β residues on average [25], are determined by V/J usage. The rest of CDR3 residues result from insertions and deletions (and D usage for TCRβ chains) at the V(D)J junction. CDR1 and CDR2 are sometimes called “germline encoded”, because their sequence is fully determined by the V genes. However, this terminology can be misleading, and it is important to emphasize that V genes are highly variable in their sequences in both chains, and that the choices of the V genes across different T cells are not germline encoded. Moreover, the majority of CDR3 residues, including several that directly contact the epitope in existing crystal structures [15], as well as the length of CDR3 sequences are heavily influenced by V and J choices [25].

TCR sequences are typically represented as Vα + CDR3α + Jα + Vβ + CDR3β + Jβ (e.g., TRAV12‐2*01, **CAV**G**DDKIIF**, TRAJ30*01, TRBV28*01, **CAS**TPQTA**YEQYF**, TRBJ2‐7*01, where the parts in bold represent amino acids coming from the V and J genes). This corresponds the most compact encoding. Alternatively, the full amino acid sequence for each chain can be used. These two encodings are equivalent in terms of information content, since there is a one‐to‐one mapping between any Vα + CDR3α + Jα + Vβ + CDR3β + Jβ and full TCR sequence. The only exception consists of TRBV6‐2 and TRBV6‐3 which have exactly the same nucleotide coding sequence and some approaches have grouped them into a single TRBV6‐2/6–3 gene, as they cannot be distinguished at the sequencing level [15, 26]. A few studies, including some aiming at modeling TCR‐epitope recognition, have proposed to use the CDR1 + CDR2 + CDR3 sequences for encoding TCRs, plus sometimes an additional region referred to as CDR2.5 [13, 27, 28]. Some loss of information arises when using this encoding since different J segments can give rise to the same CDR3 sequences. These include for instance TRBJ2‐3*01 (CDR3 part: STDTQYF) and TRBJ2‐5*01 (CDR3 part: QETQYF) if the first three, respectively first two, residues are deleted in the V(D)J recombination process. However, such cases are relatively rare, and this loss of information is unlikely to have a major impact when modeling TCR‐epitope recognition.

### 1.4 Epitope Sequence Diversity

T‐cell epitopes consist of small antigenic peptides displayed on MHC molecules. Both the peptides and the MHC molecules exhibit a very high theoretical diversity. On the peptide side, diversity arises because many amino acid positions in MHC ligands show limited specificity and MHC molecules can bind peptides of different lengths [29]. This leads to diversity estimates for peptides binding to a given MHC ranging from 10^10^ to 10^14^. On the MHC side, diversity arises because of the different genes and the high polymorphism characterizing the MHC locus. MHC diversity across humans is in the order of 10^4^ [30].

### 1.5 TCR Sequencing of T Cells With Undetermined or Known Specificity

The sequence of TCRs expressed in T cells can be determined by sequencing the two TCR loci. In bulk TCR‐sequencing, specific primers are used for each chain, and the repertoires of TCRα and TCRβ chains are sequenced separately [31, 32, 33], or only the TCRβ repertoire for some popular protocols like ImmunoSeq [34, 35]. This approach is associated with high sequencing depth and moderate costs but cannot recover actual TCRαβ pairs. Single‐cell sequencing approaches can sequence the TCRα and TCRβ chain present in each cell, thereby providing a complete picture of the paired TCRαβ repertoire of a sample [36]. Standard commercial kits are associated with a limited number of cells and high costs, but technologies are evolving fast and some approaches can now reach a very high number of cells (e.g., ParseBio or Omniscope). In parallel, studies have shown that paired TCRαβ information can be obtained by combining bulk TCRα + TCRβ sequencing of samples distributed in different wells together with computational inference of co‐occurring TCRαβ pairs [37, 38]. TCR‐sequencing has been widely applied to T cells with unknown specificity to determine the TCR repertoire of healthy donors and patients [39, 40, 41, 42]. TCR‐sequencing has also been used to sequence T cells recognizing specific epitopes [13, 43, 44, 45]. Such data have played a pivotal role in our understanding of TCR‐epitope recognition specificity [13, 15] and in the development of computational tools to predict TCR‐epitope recognition [28, 46, 47, 48, 49].

Here, we review existing approaches for TCR‐epitope recognition predictions, examine the type of data they rely on and discuss their main strengths and limitations. We then outline future steps which, in our opinion, should be taken to improve these predictions. We focus on the problem of predicting TCR‐epitope recognition using only the TCR and epitope sequences as input, since this scenario has the broadest application coverage. However, we emphasize that orthogonal information, such as clonality [50, 51], T‐cell phenotype [52, 53] or spatial location [54, 55, 56] can be very useful to narrow down the list of the most relevant antigen‐specific TCRs in biological samples.

## 2 Computational Predictions of TCR‐Epitope Recognition

Several approaches have been proposed to predict TCR‐epitope recognition [46]. They can be classified into two main categories: (1) methods that infer interaction scores directly from the TCR and epitope sequences, hereafter referred to as 1D TCR‐epitope Recognition Models (1D‐TRMs) (Figure 1B, Table 1 and Table S1), and (2) methods that rely on predicted structural 3D models to infer interaction scores, hereafter referred to as 3D TCR‐epitope Recognition Models (3D‐TRMs) (Figure 1C, Table 2 and Table S2). We emphasize that both types of approaches use as input only the TCR and epitope sequences and that the word “structure‐based” sometimes used for 3D‐TRMs simply means that these approaches use 3D structural models of the TCR‐epitope complexes predicted from the TCR and epitope sequences to assess whether the two interact.

**TABLE 1:** List of existing 1D‐TRMs with a publicly available command‐line implementation or a web interface as well as some minimal documentation.

Tool name	Epitope‐specific predictor	Pan‐epitope predictor	Epitope input type	TCR input type	Available pretrained models	Possibility to retrain	Chains that can be used in predictions
*Machine learning 1D‐TRMs*							
TEMPO [15]	✓		Peptide + MHC	(CDR3α + Vα + Jα) and/or (CDR3β + Vβ + Jβ)	✓		P/A/B
MixTCRpred [48]	✓		Peptide + MHC	CDR3α + Vα(+Jα) + CDR3β + Vβ (+Jβ)	✓		P
ERGO‐II [57]		✓	Peptide + MHC	(CDR3α + Vα + Jα) + CDR3β + (Vβ + Jβ)	✓		P/B
EPIC‐TRACE [58]		✓	Peptide + MHC	(CDR3α + Vα + Jα) and/or (CDR3β + Vβ + Jβ)	✓	✓	P/A/B
pMTnet‐omni [59]		✓	Peptide + MHC	CDR3α + Vα(+Jα) + CDR3β + Vβ (+Jβ)	✓		P
TCRAI [60]	✓		Peptide + MHC	CDR3α + Vα + Jα + CDR3β + Vβ + Jβ		✓	P
DeepTCR [61]	✓		Peptide + MHC	(CDR3α + Vα + Jα) and/or (CDR3β + Vβ + Jβ)		✓	P/A/B
NetTCR [28]	✓	✓	Peptide	CDR1α + CDR2α + CDR3α + CDR1β + CDR2β + CDR3β	✓	✓	P
TCRGP [62]	✓		Peptide	(CDR1α + CDR2α) + CDR3α and/or (CDR1β + CDR2β) + CDR3β	✓	✓	P/A/B
Tspred [63]		✓	Peptide	CDR1α + CDR2α + CDR3α + CDR1β + CDR2β + CDR3β	✓	✓	P
TULIP [49]		✓	Peptide + MHC	CDR3α and/or CDR3β	✓	✓	P/A/B
TCR‐BERT [64]	✓		Peptide	CDR3α and/or CDR3β	✓	✓	P/A/B
STAPLER [65]		✓	Peptide	CDR3α + CDR3β	✓	✓	P
DLpTCR [66]		✓	Peptide	CDR3α and/or CDR3β	✓	✓	P/A/B
TCRconv [67]	✓		Peptide	CDR3α and/or CDR3β	✓	✓	P/A/B
TCR‐pred [68]	✓		Peptide	CDR3α and/or CDR3β	✓		P/A/B
TCRex [69]	✓		Peptide	CDR3β (+ Vβ + Jβ)	✓	✓	B
TITAN [70]		✓	Peptide	CDR3β (+ TCRβ)	✓	✓	B
TPBTE [71]		✓	Peptide	CDR3β		✓	B
pMTnet [72]		✓	Peptide + MHC	CDR3β	✓		B
PISTE [73]		✓	Peptide + MHC	CDR3β	✓	✓	B
epiTCR [74]		✓	Peptide (+MHC)	CDR3β	✓	✓	B
ImRex [75]		✓	Peptide	CDR3β	✓		B
ATM‐TCR [76]		✓	Peptide	CDR3β	✓		B
TEIM [77]		✓	Peptide	CDR3β	✓	✓	B
TEPCAM [78]		✓	Peptide	CDR3β	✓	✓	B
TEInet [79]		✓	Peptide	CDR3β	✓	✓	B
vitTCR [80]		✓	Peptide	CDR3β		✓	B
Attn‐TAP [81]		✓	Peptide	CDR3β	✓	✓	B
iTCep [82]		✓	Peptide	CDR3β	✓		B
SETE [83]	✓		Peptide	CDR3β	✓	✓	B
PiTE [84]		✓	Peptide	CDR3β		✓	B
PanPep [85]		✓	Peptide	CDR3β	✓	✓	B
TCR‐epiDiff [86]		✓	Peptide	CDR3β	✓		B
UniPMT [87]		✓	Peptide	CDR3β	✓		B
GTE [88]		✓	Peptide	CDR3β		✓	B
LANTERN [89]		✓	Peptide	CDR3β	✓	✓	B
TCR‐H [90]		✓	Peptide	CDR3β		✓	B
EpicPred [91]	✓		Peptide	CDR3β		✓	B
TpepRet [92]		✓	Peptide	CDR3β	✓	✓	B
tcrLM [93]		✓	Peptide	CDR3β	✓	✓	B
DeepPROTECTNeo [94]		✓	Peptide	CDR3β		✓	B
DAISY [95]		✓	Peptide	CDR3β	✓	✓	B
proTCR [96]		✓	Peptide	CDR3β		✓	B
TCRfinder [97]		✓	Peptide	CDR3β	✓		B
PepTCR‐Net [98]		✓	Peptide	CDR3β	✓		B
*Distance‐based 1D‐TRMs*							
TCRdist3 [99]	✓		NA	CDR3α + Vα + Jα + CDR3β + Vβ + Jβ		✓	P/A/B
SCEPTR [100]	✓		NA	CDR3α + Vα(+Jα) + CDR3β + Vβ (+Jβ)		✓	P/A/B
TCRbase [101]	✓		NA	CDR1α + CDR2α + CDR3α + CDR1β + CDR2β + CDR3β		✓	P
GLIPH2 [102]	✓		NA	(CDR3α+)CDR3β + Vβ(+Jβ)		✓	P/B
SwarmTCR [103]	✓		peptide	(CDR1α + CDR2α + CDR2.5α + CDR3α) and/or (CDR1β + CDR2β + CDR2.5β + CDR3β)	✓	✓	P/A/B
TCRMatch [104]	✓		peptide	CDR3β	✓		B

*Note:* Parentheses indicate optional features. In the last column, “P” stands for paired TCRαβ sequences, “A” stands TCRα sequences and “B” stands for TCRβ sequences. The list may not be complete, and we apologize if we have overlooked some tools. Additional information for each tool is available in Table S1.

**TABLE 2:** List of existing 3D‐TRMs with a publicly available command‐line implementation or web interface as well as some minimal documentation.

Tool name	Epitope input type	TCR input type	Input	Underlying models	Binding evaluation	Chains that can be used in predictions
*Methods relying on confidence metrics*						
TCRDock [105]	Peptide + MHC	CDR3α + Vα + Jα + CDR3β + Vβ + Jβ	Sequence	AF2	AF confidence metrics	P
TCRBridge [106]	Peptide + MHC	CDR3α + Vα + Jα + CDR3β + Vβ + Jβ	3D model	AF3	AF confidence metrics	P
HERMES [107]	Peptide + MHC	CDR3α + Vα + Jα + CDR3β + Vβ + Jβ	3D model	HERMES	Structure‐based neural network	P
tFold‐TCR [108]	Peptide + MHC	CDR3α + Vα + Jα + CDR3β + Vβ + Jβ	Sequence	ESM2	Model confidence metrics	P
*Custom structural descriptors or interface scoring functions*						
NetTCR‐struc [109]	Peptide + MHC	CDR3α + Vα + Jα + CDR3β + Vβ + Jβ	3D model	AF‐multimer	GNN‐predicted DockQ scores	P
TCRen [110]	Peptide + MHC	CDR3α + Vα + Jα + CDR3β + Vβ + Jβ	3D model	N/A	Contact‐based scores	P
TCRlens [111]	Peptide + MHC	CDR3α + Vα + Jα + CDR3β + Vβ + Jβ	3D model	N/A	GNN‐predicted binding	P
t2pmhc [112]	Peptide + MHC	CDR3α + Vα + Jα + CDR3β + Vβ + Jβ	Sequence	TCRDock (AF2)	GNN‐predicted binding	P
TCRcost [113]	Peptide	CDR3α + CDR3β	3D model	AF2	3D‐CNN‐predicted binding	P
*Multimodal deep learning frameworks*						
DeepAIR [114]	Peptide + MHC	CDR3α + Vα + Jα + CDR3β + Vβ + Jβ	Sequence +3D model	AF2	Multimodal deep learning framework	P
STAG‐LLM [115]	Peptide + MHC	CDR3α + Vα + Jα + CDR3β + Vβ + Jβ	Sequence +3D model	ESM2/AF2	Multimodal deep learning framework	P
SageTCR [116]	Peptide + MHC	CDR3α + Vα + Jα + CDR3β + Vβ + Jβ	3D model	ChemBERTa/Saprot	Multimodal deep learning framework	P
StriMap [117]	Peptide + MHC	CDR3α + Vα + Jα + CDR3β + Vβ + Jβ	Sequence	ESM2/ESMFold	Multimodal deep learning framework	P
TCRcube [118]	Peptide + MHC	CDR3α + CDR3β	Sequence	ESM2/AF2	Multimodal deep learning framework	P
TRAP [119]	Peptide + MHC	CDR3β	Sequence +3D model	ESM2/AF‐Multimer	Multimodal deep learning framework	B
CATCR [120]	Peptide	CDR3β	Sequence +3D model	OpenFold	Multimodal deep learning framework	B

*Note:* In the last column, “P” stands for paired TCRαβ sequences and “B > TABLE” stands for TCRβ sequences. The list may not be complete, and we apologize if we have overlooked some tools. Additional information for each tool is available in Table S2.

Abbreviations: CNN, convolutional neural network; GNN, graph neural network.

### 2.1 Methods Inferring Interaction Scores Directly From the TCR and Epitope Sequences (1D‐TRMs)

The availability of easy‐to‐download and well‐structured databases of TCRs recognizing different epitopes [121, 122, 123] has motivated the development of multiple tools to leverage these data and build 1D‐TRMs to predict TCR‐epitope recognition. To review dominant patterns in existing tools, we performed a survey of several methods, using as selection criteria the availability of a command‐line implementation or a web interface as well as some minimal documentation (Table 1 and Table S1). Two main classes of 1D‐TRMs can be outlined (Figure 1B). First, machine learning 1D‐TRMs aim at embedding the TCR (and often the epitope) sequences into some high‐dimensional space and learning a possibly very complex function or manifold that characterizes TCRs recognizing each epitope. Such models can then score a new TCR‐epitope pair to predict whether they interact or not. Second, distance‐based 1D‐TRMs aim at using distance or similarity measures between TCRs to quantify how similar a new TCR is to TCRs known to interact with a given epitope, and predict the interaction with this epitope based on this similarity. Both machine learning tools and distance‐based approaches are computationally efficient to annotate large TCR repertoires and can score from a few thousand to a million TCRs per minute on a standard desktop [15].

#### 2.1.1 Machine Learning Approaches

These are the most widely used approaches to predict TCR‐epitope interactions, and close to 50 different machine learning 1D‐TRMs have been proposed in recent years. Some use directly the TCR sequences and learn some latent space in which a classifier is built, while others rely on protein language models [64]. Several patterns emerge in terms of the scope of the predictions and the features that are considered. For instance, the vast majority of tools can, in principle, make predictions for any epitope (Figure 1D and Table 1). These are referred to as *pan‐epitope*. Many studies describing such tools have claimed generalizability to unseen epitopes (i.e., epitopes without known TCRs) [49, 58, 90, 124]. However, these claims could not be confirmed in recent benchmarks [45, 125, 126, 127, 128]. Other tools provide separate models for each epitope and are referred to as *epitope‐specific* [15, 48, 62, 69]. Some tools combine pan‐epitope and epitope‐specific architectures [28]. Data used to train these tools come mainly from three databases: VDJdb [129], IEDB [122], and McPAS [123]. Different studies using these data to train 1D‐TRMs include different levels of data curation (see discussion below).

Despite the large number of machine learning 1D‐TRMs, only five of them (i.e., TEMPO [15], MixTCRpred [48], ERGO‐II [57], EPIC‐TRACE [58], and pMTnet‐omni [59]) provide pretrained models that consider the full TCR and the full epitope sequences (i.e., Vα + CDR3α + Jα + Vβ + CDR3β + Jβ and peptide+MHC) (Figure 1E and Table 1). A few other methods do not consider the MHC [28, 62, 63]. While this may lead to some confusion since the same peptide presented on different MHCs can be recognized by very distinct TCRs [15, 130], the original MHC allele can be retrieved relatively easily in most cases. Most other tools focus on CDR3 sequences, with many of them considering only the CDR3β sequence and often abusively referring to it as the “TCR sequence”. This likely represents an important limitation, since the specificity encoded in the alpha chain and much of the specificity encoded in Vβ usage cannot be inferred from CDR3β sequences [15, 131].

#### 2.1.2 Distance‐Based Approaches

These approaches rely on similarity or distance measures between TCRs to infer whether a TCR binds to a given epitope by computing its distance to TCRs known to bind to this epitope (Table 1). Some of these approaches, such as TCRdist [13], use general biological/structural knowledge to define the distances. Others use general protein language models such as ESM‐2 or ProtBert, or models trained specifically for TCRs [100]. Many of these tools rely on the nearest‐neighbor principle to make predictions of epitope restriction, and often require users to provide a set of TCRs annotated with their cognate epitope that is used as a reference for the distance calculations. Recent independent benchmarks suggest that different distance definitions, including basic metrics such as CDR edit distance, lead to similar prediction accuracy [45]. Some approaches consider the full TCR sequences or the CDR loops for both chains, while others focus on subparts (e.g., CDR3α + CDR3β + Vβ + Jβ for GLIPH2 [43] or CDR3β only for TCRMatch [104]). All these approaches fall into the epitope‐specific category since they can only make predictions for epitopes with known TCRs. Some provide annotated reference datasets of epitope‐specific TCRs, while others require users to provide reference data when using the tools for TCR‐epitope interaction predictions. As they do not attempt to learn complex functions that require large training data, they can be applied to epitopes with few known TCRs, although prediction accuracy is higher for epitopes with many known TCRs. Distance‐based approaches can be sensitive to contaminants in the data and most of them do not learn which parts of the TCR sequences encode more or less specificity for a given epitope.

### 2.2 Methods Relying on Structural Modeling (3D‐TRMs)

Another class of models aims at exploiting information encoded in predicted structural models of TCR‐epitope complexes. Historically, limited success was met with such approaches, mainly because of the challenges to obtain reliable models of TCR‐peptide–MHC (pMHC) 3D complexes besides cases of very high homology with an experimental TCR‐pMHC crystal structure. However, the emergence of deep learning‐based protein structure prediction methods, including AlphaFold2 (AF2) [132], its successor AlphaFold3 (AF3) [16], and related architectures [133, 134], has dramatically improved modeling accuracy at the TCR–pMHC interface, enabling informative interaction analysis even without closely related structural templates [108, 128, 135]. Nevertheless, accurately resolving TCR–pMHC binding geometry remains challenging in many cases, owing to weak inter‐chain co‐evolutionary signals, highly flexible CDR loops, and the limited availability of experimentally resolved TCR–pMHC complex 3D structures. Several strategies have been proposed to enhance performance, including the use of custom structural templates, tailored multiple sequence alignments, and model fine‐tuning on TCR–pMHC‐specific datasets [105]. Beyond improving structural prediction itself, diverse strategies have been developed to extract interaction‐relevant signals from predicted TCR–pMHC complexes. These approaches can be broadly grouped into three paradigms (Table 2). First, some approaches rely on intrinsic confidence metrics provided by structure predictors, such as Interface Predicted Template Modeling (ipTM) or Predicted Aligned Error (PAE), as proxies for interaction likelihood, like TCRBridge or TCRDock [105, 106]. Second, custom structural descriptors or interface scoring functions convert predicted coordinates into energy‐like or contact‐based scores, like in TCRen or NetTCR‐struct [109, 110]. Third, multimodal deep learning frameworks integrate structure‐derived embeddings with sequence information to learn interaction patterns directly, such as DeepAir, STAG‐LLM [114, 115]. Each paradigm reflects a distinct trade‐off between interpretability, generalization capacity, and robustness to geometric uncertainty.

While the field has not yet reached full maturity, many early reports provide clear evidence of remarkably successful predictions for some epitopes, including cases of TCRs binding to unseen epitopes that show no sequence similarity with epitopes with known TCRs [18, 105, 106, 109]. This was confirmed by the results of the IMMREP25 competition where TCRs binding to epitopes without known TCRs in public databases were isolated and the task was to predict which TCRs bind to which epitopes [128]. Consistent with previous benchmarks [45, 126], all 1D‐TRMs failed, whereas some predictive power was observed for some epitopes with methods using 3D models of TCR‐epitope complexes to score the interactions. It is therefore expected that such approaches will have an important impact on TCR‐epitope recognition predictions. As of today, one important challenge is to know when predictions of 3D‐TRMs can be trusted and when they fail, and why this is the case. Another limitation of 3D‐TRMs relates to computational efficiency. Typically, modeling and scoring one TCR‐epitope complex can take from a few tens of seconds to several minutes [105, 106, 136], which makes it challenging to score repertoires of hundreds of thousands of TCRs across cohorts of thousands of patients for dozens of epitopes [42].

## 3 The Challenges With the Positives

All 1D‐TRMs rely on training data in the form of interacting TCR‐epitope pairs, either for fitting the scoring function used in machine learning predictors or for computing distances (Figure 1B). As in many other fields, the quality and depth of the training data play a central role in the accuracy of the predictions. Moreover, all 1D‐ and 3D‐TRMs rely on such data for benchmarking predictions.

**FIGURE 2:**
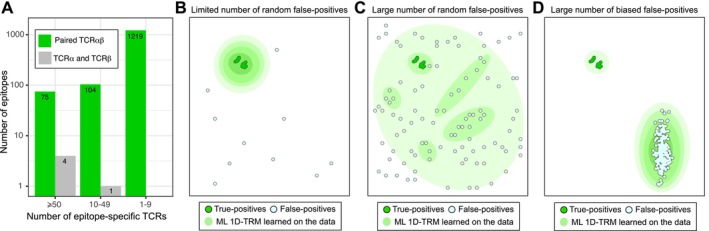
(A) Number of epitopes with high (≥ 50), modest (10–49) or limited (1–9) number of epitope‐specific TCRs. The green bars show the statistics counting paired TCRαβ only. The gray bars show the number of epitopes which have lower number of paired TCRαβ than the different thresholds but higher number of unpaired TCRα and TCRβ sequences. (B) Illustration with the green shaded ellipses of a typical machine learning 1D‐TRM in the presence of a limited number of random false positives in the training data. (C) Illustration with the green shaded ellipses of a typical machine learning 1D‐TRM in the presence of a large number of random false positives in the training data. (D) Illustration with the green shaded ellipses of a typical machine learning 1D‐TRM in the presence of a large number of biased false positives in the training data, such as TCRs binding to another epitope. We emphasize that cartoons in panels B–D aim at providing an interpretable visualization of different scenarios that can be encountered in the training data of machine learning 1D‐TRMs, but do not capture the high‐dimensionality of the TCR sequence space.

For TCR‐epitope interactions, abundant (i.e., ≥ 50 TCRs for both chains) data is available for 79 epitopes based on our latest internal compilation of existing databases and recent studies [15, 121, 122, 126, 128, 137] (Figure 2A). Such data have been instrumental for understanding key principles of TCR‐epitope recognition specificity, including specific enrichment in V/J genes, CDR3 lengths and CDR3 sequence motifs across different epitopes [13, 15, 43]. Beside these cases, some data (i.e., 10–49 TCRs) is available for a bit more than one hundred epitopes, very limited data (i.e., 1–9 TCRs) is available for a bit more than one thousand epitopes (Figure 2A), and no training data is available for the tens of thousands of other possible, often unknown or poorly characterized, epitopes in infectious diseases, cancer or autoimmunity. This very limited coverage of the epitope space explains why 1D‐TRMs have failed to extrapolate to unseen epitopes [75, 128, 138]. Recent studies have highlighted important issues with the quality of existing data [25, 48, 106]. We discuss below some of the main issues that can be found in datasets of epitope‐specific TCRs. However, we emphasize that many studies are not impacted by these issues and provide reliable data that can be used to train or benchmark TCR‐epitope interaction predictors.

### 3.1 Isolation of Epitope‐Specific T Cells

The experimental identification of TCRs binding to specific epitopes typically relies on the isolation of T cells recognizing an epitope and the sequencing of their TCRs. Most often, this isolation is done by sorting T cells with flow cytometry, using pMHC multimers or antibodies recognizing T‐cell activation surface markers like CD137. The TCRs in those T cells can then be determined with the TCR‐sequencing pipelines discussed in the introduction. Several issues can arise in these experiments.

#### 3.1.1 Issues With Experimental Design and Negative Controls

Although flow cytometry is a mature technology, careful negative controls are needed to minimize the risk of false positives when isolating epitope‐specific T cells. Depending on the studies, such controls may not have always been performed, which may result in cases where T cells are reported to recognize a specific epitope while they correspond to background noise. Issues with gating strategies can also arise and are sometimes difficult to trace back. These risks are especially important in large‐scale studies aiming at collecting big datasets of TCRs recognizing multiple epitopes, since verifying all parameters used in the analyses for each epitope can be difficult.

#### 3.1.2 Issues With pMHC Multimers

The construction of pMHC multimers requires specialized skills and is challenging for some classes of MHC molecules like MHC‐II. In theory, pMHC multimers should always be validated with known epitope‐specific TCRs. This validation is possible when working with well‐studied epitopes. However, when considering novel epitopes, positive controls are much more difficult to establish. It is likely that some reported epitope‐specific TCRs that could not be validated in subsequent studies resulted from issues in the pMHC multimers used in the initial study.

To improve the throughput of pMHC multimer‐based annotation of epitope‐specific T cells, studies have designed DNA‐barcoded multimers which can be used to stain heterogeneous T‐cell populations [139, 140]. Single‐cell TCR/barcode sequencing can then be applied to infer pairs of interacting TCRs and epitopes. A challenge with this approach comes from DNA‐barcode leakage. For instance, in a large dataset made available by 10X Genomics [141], multiple barcodes have been observed in many cells, leading to a substantial level of false positives, as reported by multiple studies [15, 28, 48].

#### 3.1.3 Issues With Expression of Activation Markers

An alternative to sorting with pMHC multimers consists of stimulating isolated peripheral blood mononuclear cells with a specific peptide and sorting T cells based on (over‐)expression of activation markers, such as CD137 [106, 142]. This approach has the advantage of using the same set of antibodies for all epitopes. However, here again, some issues can arise. First, it cannot be determined with 100% certainty on which MHC allele the peptide used in the stimulation was actually displayed. This issue is especially relevant if the cells used in the stimulation harbor MHC molecules with some similarity in their binding motifs [29]. Second, one cannot exclude that a shorter version of the peptide used for stimulation is actually presented on some MHC since these peptides can be cleaved during antigen processing. This issue is especially relevant when using 10‐mer or longer peptides presented on MHC‐I molecules since the shorter version of these peptides can sometimes bind to the same or other MHCs. Finally, unspecific activation, although relatively rare, cannot be excluded.

In addition to quality controls in the experiments, we strongly encourage researchers to always visualize any newly generated dataset of epitope‐specific TCRs to detect signs of potential contaminations. To this end, the TCR specificity profile framework recently developed in our group [15] provides a standardized way for seamlessly exploring repertoires of epitope‐specific TCRs (see https://tcrmotifatlas.unil.ch/Building_motifs). This framework enables rapid comparison with other datasets of TCRs recognizing the same epitope when such data is available. It can also reveal lack of specificity characterized by V/J gene usage and CDR3 length distributions that follow those of baseline TCR repertoires. Such cases are often associated with a high number of false positives in the data [15].

### 3.2 TCR‐Sequencing and TCR Reconstruction

#### 3.2.1 Issues With TCR‐Sequencing

All TCR sequencing protocols are prone to errors. Such errors can occur either during the PCR amplification or during the sequencing itself. PCR errors are relevant for many TCR‐sequencing protocols that capitalize on multiple cycles of PCR amplification to better detect reads from the TCR loci. Errors in the sequencing are rare with today's instruments, but the high number of reads that are sequenced makes such errors still possible. PCR and sequencing errors can give rise to CDR3 sequences with the wrong amino acids or challenges in reconstructing V/J genes. Approaches to mitigate these issues capitalize on unique molecular identifiers and statistical models have been developed to remove many of the spurious sequences [143].

#### 3.2.2 Issues With TCR Reconstruction

When sequencing TCRs, reads only capture subparts of the TCR sequences, which typically include the full CDR3 + adjacent nucleotides, and sometimes other subparts of the V segments. The actual full TCR sequences are then determined by inferring the V and J genes from the sequencing reads. Some V genes show high sequence similarity, which can lead to ambiguities. These include for instance TRBV6‐2 and TRBV6‐3 which have exactly the same nucleotide sequence or TRBV12‐3 and TRBV12‐4 which have identical sequence for the last 199 nucleotides. Such cases can be difficult, or impossible in the case of TRBV6‐2 and TRBV6‐3, to distinguish when reconstructing TCRs from TCR‐sequencing data. In other cases, other types of errors have been made when inferring V and J segments, especially in older datasets. Some of these issues can be detected by checking the compatibility between CDR3 sequences and V/J genes [25], although this approach cannot find all possible issues due to the similarity in the CDR3 part of many V genes, such as the CASS motif in most Vβ genes in human.

### 3.3 Data Processing and Deposition

#### 3.3.1 Issues With TCR Sequences

Many issues have been observed in TCR sequences deposited in databases. One common problem comes from different definition of CDR3s. Typically, most studies use a definition of CDR3 starting with the conserved cysteine (C) and ending in human at the conserved phenylalanine (F) for most J genes, tryptophan (W) for TRAJ33, TRAJ38 and TRAJ55, or cysteine (C) for TRAJ35. However, other studies have dropped the first and last amino acids. Unfortunately, different definition of CDR3s have sometimes been used even within the same study [144]. Other issues occur regarding V and J gene nomenclature. This is because different studies have used different naming conventions. For instance, the TRAV1‐2 gene (IMGT nomenclature) is sometimes referred to TCRAV1‐2, hTRAV1‐2, TRAV01‐2, TRAV01‐02, Va1‐2 or TRA1‐2. In addition, some studies include allelic information (TRAV1‐2*01) while others report only gene level annotation (TRAV1‐2). While approaches have been designed to harmonize datasets [25, 145, 146], complete harmonization is still challenging because some differences are quite counter‐intuitive. For instance, TCRBV12‐1 in the gene nomenclature used by Adaptive Biotechnologies actually corresponds to TRBV12‐3 in the IMGT nomenclature, but TRBV12‐1 exists in IMGT, albeit encoding a pseudogene [30]. Similarly, TCRBV7‐1 in Adaptive Biotech is TRBV7‐6 in IMGT. We advocate to use the nomenclature defined in the IMGT database [24], which is the most widely used throughout existing studies and databases, and has been carefully maintained over many decades. Guidelines provided by the AIRR community are also very useful to improve standardization across datasets (https://docs.airr‐community.org). Other types of errors can occur due to inappropriate processing of the data. For instance, in the McPAS database [123], a recent study by our group reported that the second digit of several V and J gene names had been dropped (e.g., TRBV1‐01 instead of TRBV19*01, or TRAJ4‐01 instead of TRAJ42*01) [25]. These different issues likely contributed to the decision in many studies to overlook V and J genes and to only consider CDR3 sequences when modeling TCR‐epitope recognition specificity (Figure 1E), despite the central role in epitope recognition played by residues outside of CDR3s [11, 14, 15].

#### 3.3.2 Issues in Peptide Sequences

Inaccuracies in peptide sequences can also happen. First, as already mentioned, in some cases a peptide used for stimulation has been cleaved and some TCRs reported to recognize the initial peptide actually recognize a shorter version of this peptide. In other cases, manual errors occur when building files to upload data in databases. For instance, in one study [147], the Melan‐A epitope (ELAGIGILTV restricted to HLA‐A*02:01) was used, but TCRs were entered as binding to ELAGIGLTV (i.e., second I missing before LTV) in VDJdb [121]. Many studies also use the three first letters of the peptide sequences (e.g., ELA for ELAGIGILTV). While this makes it much easier for human brains to remember and, in most cases, unambiguously identifies the different peptides used in a study, it can create issues when integrating data from multiple studies.

#### 3.3.3 Issues in MHC Sequences

Several issues are also encountered with MHC alleles. First, many studies do not report the MHC. This can be the case when the MHC restriction cannot be unambiguously determined, for instance when using peptide stimulation followed by sorting based on expression of activation markers. Unfortunately, even when the MHC was known, some studies only provide the peptide sequences. For common epitopes, the MHC restriction can often be guessed, but this is not always the case. Issues also arise with different naming of the MHC alleles. For instance, the well‐studied HLA‐A*02:01 allele appears as A*02:01, A0201, A2, HLA‐A*02, HLA‐A2, HLA‐A*0201, HLA‐A*02:01:110, HLA‐A*02:01:48, HLA‐A*02:256 or HLA‐A*02:266 across different studies in databases of epitope‐specific TCRs. Similar ambiguities occur frequently with mouse MHC alleles. For instance, H2‐Kb, H‐2KB or H‐2Kb refer all to the same mouse MHC. These issues can be challenging to correct and likely contributed to the decision in many studies modeling TCR‐epitope recognition to overlook the MHC in their models (Figure 1E), despite the central role played by interactions between TCR and MHC residues in epitope recognition.

The presence of a substantial fraction of false positives has been experimentally demonstrated in a recent study for a few epitopes with many reported TCRs [106]. The consequences on TCR‐epitope recognition predictions of such errors differ depending on the applications. When using such data for benchmarking 1D‐TRMs or 3D‐TRMs, false positives can lower the apparent predictive power of specific tools, and even highly accurate approaches may give rise to only modest Area Under the receiver operating characteristic Curve (AUC) values. When using such data for training 1D‐TRMs, the impact will differ depending on the amount and the type of contaminations. Some limited and relatively random contaminations are unlikely to impact much machine learning 1D‐TRMs, since most of them can tolerate some false positives in their training data [15] (Figure 2B). Distance‐based 1D‐TRMs can be more sensitive to such contaminants. Extensive random contaminations can have a big impact on 1D‐TRMs and often result in predictions that show limited specificity across the TCR space and lots of false positives in the top predictions (Figure 2C). Finally, contaminations showing strong biases, such as specificity for another epitope, can significantly bias a model (Figure 2D).

### 3.4 The Challenges With the Negatives

Most TCR‐epitope recognition prediction approaches require some information about negatives, defined as TCRs that do not bind the epitope. For instance, most machine learning 1D‐TRMs either explicitly use negatives in the training data [27, 28, 48], or implicitly assume that TCR‐epitope combinations absent from the positives do not bind [49]. Distance‐based 1D‐TRMs often rely on negatives to define reasonable thresholds on the distance or similarity measures. Benchmark datasets for 1D‐TRMs or 3D‐TRMs also require non‐binding TCR‐epitope pairs to compute performance metrics like AUC. In the case of TCR‐epitope recognition, few experimentally validated negatives are known. Moreover, these data are often biased, including, for instance, point mutations in CDR3 loops or in epitope sequences, and do not reflect the full diversity of non‐binding TCR‐epitope pairs. For this reason, most studies have used artificially generated negatives, and different approaches have been proposed.

**FIGURE 3:**
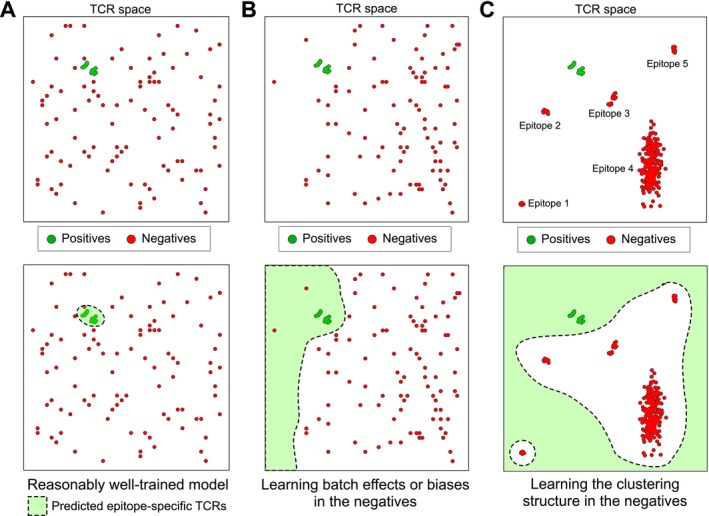
(A) Example of random negatives that cover adequately the diversity of native TCR repertoires. Below is an illustration of a possible 1D‐TRM trained on these data that gives rise to reasonable, though not perfect, predictions. (B) Example of random negatives that contain substantial batch effects/biases. Below is an illustration of the risk for a 1D‐TRM to learn such biases, which could result in apparent high prediction accuracy if the same biases appear in the negatives used in training and test sets, which is typically the case in standard cross‐validation settings. (C) Example of swapped negatives coming from a few other epitopes, including one with many TCRs (Epitope 4). Below is an illustration of the risk for a 1D‐TRM to learn the specificity and the clustering structure of the negatives, which could result in apparent high prediction accuracy if TCRs binding to the same epitopes appear as negatives in the training and test sets. We emphasize that these cartoons aim at providing an interpretable visualization of different scenarios that can be encountered in machine learning 1D‐TRMs, but do not capture the high‐dimensionality of the TCR sequence space.

One possible approach, referred to as “random negatives”, is to randomly select TCRs from repertoires of TCRs with undetermined specificity (Figure 3A). This approach mimics realistic scenarios where epitope‐specific TCRs are to be predicted from TCR repertoires sequenced in patients or healthy donors. The risk of false negatives is small, since only a very tiny fraction of TCRs found in TCR repertoires of undetermined specificity bind to a given epitope. One important issue can emerge when using the same or related studies for the negatives in both training and testing data if these studies display important batch effects or biases compared to the studies used to generate the positives (see illustration in Figure 3B). These biases typically impact V/J usage, which will in turn result in different length and amino acid composition of CDR3 sequences [25]. If this is the case, any machine learning algorithm is at high risk of learning such patterns, which can result in artificially inflated prediction accuracy and even apparent predictive power on unseen epitopes [148].

Another approach, which has been used in many studies and is often referred to as “swapped negatives”, consists of taking as negatives some TCRs reported to bind to other epitopes (Figure 3C) [57, 148, 149]. The main motivation for this approach was to address the previously mentioned risk of batch effects or biases between positives and negatives [148]. As with random negatives, the likelihood of having false negatives is limited and will in general not impact much the predictions. However, other issues emerge with the swapped negatives approach. First, this scenario does not reflect realistic applications of TCR‐epitope interaction predictions, since one rarely faces the task of annotating repertoires consisting only of TCRs recognizing a few dozen predetermined and well‐studied epitopes from mixed origins, like common viruses and cancer epitopes. Second, batch effects or biases may still exist if the TCRs specific for the different epitopes come from different studies, which is typically the case when considering large collections of epitope‐specific TCRs used to train most existing tools. Third, because some epitopes used to derive negatives have many known TCRs, such as A0201_GILGFVFTL, significant specificity patterns often appear in the negatives [15]. Fourth, this approach creates a strong clustering structure in the negatives. Machine learning algorithms are at high risk of learning these potential batch effects, specificity patterns or clustering structures in the negatives. This can result in significantly inflated prediction accuracy, including apparent predictive power for unseen epitopes. Approaches proposed to mitigate this include using the partial AUC at 10% false‐positive rate (AUC01) instead of the standard AUC, which lowers the risk of inflated AUC obtained by “learning the negatives,” but does not completely prevent it.

Because swapped negatives do not recapitulate realistic applications of TCR‐epitope recognition predictions, we advocate not to use them for benchmarking existing tools and instead use TCRs from TCR repertoires. In this case, TCRs used as negatives should ideally come from the same study and same donor as the positives. If this is not practically feasible, different studies should be used to collect negatives in the training and testing data, and efforts should be made to ensure that these studies do not share similar batch effects. In our hands, the framework of TCR specificity Profile represents a convenient tool for rapidly exploring putative biases across studies [15]. Should the swapped negatives strategy still be selected, we strongly advocate to make sure that negatives in the training and test sets do not come from the same epitopes so that 1D‐TRMs cannot learn specificity or clustering patterns in negatives that are shared between the training and test data.

Beside the risk of biases in the negatives, it is also important to realize that even a large excess of negatives compared to the number of positives in the training data of 1D‐TRMs will only cover a tiny fraction of the full theoretical diversity of TCR repertoires. As proposed in our recent work [15], approaches relying on probabilistic models of baseline TCR repertoires trained on hundreds of millions of TCR sequences and used for modeling negatives represent a promising alternative that overcomes the inherent limitations encountered when explicitly providing negatives for training 1D‐TRMs.

## 4 Modeling TCR‐Epitope Recognition Specificity—Why the Size of the TCR and Epitope Sequence Space Matters

The enormous theoretical diversity in both TCR and epitope sequences has a profound impact on how to model TCR‐epitope recognition specificity.

**FIGURE 4:**
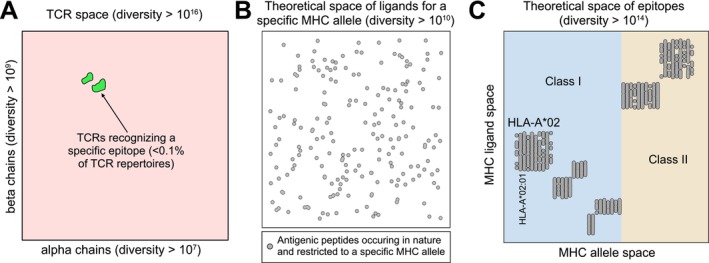
(A) Illustration of the relative size and theoretical diversity of repertoires of TCRs of undetermined specificity (red) and those recognizing a specific epitope (green). (B) Illustration of the diversity and absence of evolutionary relationship among antigenic peptides found in nature and restricted to a specific MHC within the theoretical space of ligands of this MHC allele. (C) Illustration of the structure of the epitope (i.e., Peptide + MHC) space, with a clear clustering structure induced by the evolutionary relationships among MHC alleles which occur in families (e.g., HLA‐A*02 alleles) with similar binding motifs.

On the TCR side, the high theoretical diversity of TCR sequences (> 10^16^) and the high specificity observed in TCR‐epitope recognition indicate that only a very tiny fraction of all existing TCRs can recognize a given epitope (Figure 4A). This implies that the likelihood of having TCRs that show strong similarity in their sequence with TCRs recognizing a specific epitope but do not bind to this epitope is actually quite small in realistic repertoires from patients. These cases include swapped alpha–beta pairs which do not bind to an epitope despite having seemingly good alpha and good beta chains. Moreover, such non‐binding TCRs are unlikely to be selected and undergo clonal expansion in vivo, since most of them would not bind any epitope present in a patient.

The diversity in both alpha and beta chains, and their fundamental role in epitope recognition, further suggests that information about full TCR sequences (i.e., Vα + Jα + CDR3α + Vβ + Jβ + CDR3β) should ideally be considered when predicting TCR‐epitope recognition. This is because, for most epitopes, a chain compatible with the binding to the epitope is much more likely to occur with a complementary chain that prevents binding to the epitope than with a complementary chain that preserves the binding to the epitope. Exceptions to this principle include TCRs recognizing dominant shared epitopes, or TCRs expressed in specific subtypes of T cells like mucosal‐associated invariant T (MAIT) cells. Another important consideration arises when benchmarking methods that only consider single chain TCRs. Because experimentally validated paired TCRαβ sequences are used in such benchmarks, most single‐chain TCRs considered as positives are paired with a complementary chain that is compatible with recognition of the same epitope. However, this is not the case in actual repertoires, as discussed above. This can result in inflated sensitivity when evaluating methods that consider only one chain, since most of the positives would actually not bind if paired with non‐predetermined good complementary chains. In other words, single‐chain TCRs used as positives in current benchmarks are actually much more likely to occur as non‐binders in realistic TCR repertoires due to their probable pairing with very diverse, and most often non‐binding, complementary chains.

The size and diversity of the epitope space also have important consequences on modeling TCR‐epitope recognition. To illustrate this aspect, let us first consider all antigenic peptides binding to a specific MHC allele. Such peptides can come from hundreds of highly diverse pathogens, from tumor‐associated antigens or cancer‐specific non‐synonymous alterations, and even from self‐proteins in autoimmune diseases. As a result, most of them do not share any homology beyond that imposed by the binding to the MHC. The general lack of similarity between most antigenic peptides found in nature besides the one imposed by similar MHC restriction and by amino acid baseline frequencies indicates that such peptides span uniformly the space of potential MHC ligands and cannot be grouped into a few clusters or “epitope supertypes” (see illustration in Figure 4B). This suggests that most naturally occurring epitopes will not share similar TCR recognition rules. This hypothesis has been broadly confirmed in experimental data, where limited similarity has been observed in TCRs recognizing distinct peptides restricted to the same MHC [15]. The only exceptions consist of some peptides that display very high sequence similarity. These cases can include closely related variants observed in different viral strains [150] or artificial sequence modifications (e.g., EAAGIGILTV **→** ELAGIGILTV in MAGEA‐3 [15] or ITDQVPFSV **→** IMDQVPFSV in PMEL [124]). In line with recent studies [75, 138], this observation highlights the challenges faced when attempting to extrapolate TCR‐epitope recognition rules across the space of peptides, even when fixing the MHC restriction.

Some structure emerges in epitope space when considering the MHC because of the evolutionary relationship between MHC alleles that can be grouped in families (e.g., HLA‐A*02 genes) of highly related alleles (Figure 4C). In some cases, the same peptide presented on two highly similar MHC molecules leads to conserved TCR recognition (e.g., HLA‐A*02:01 and HLA‐A*02:06) [15, 151]. However this is not always the case and even relatively small changes (e.g., HLA‐A*02:01 → HLA‐A*02:05) have been reported to impact TCR recognition [15, 152]. More significant changes in MHC restriction (e.g., HLA‐A*02:01 → HLA‐B*13:02 for LLWNGMPAV [15] or HLA‐B*07:02 → HLA‐B*08:01 for RPIIRPATL [130]) have always been associated with recognition by distinct TCRs.

Overall, these observations emphasize how the size and complexity of the TCR and epitope sequence spaces, which have been discussed in many previous studies, have important consequences when modeling TCR‐epitope recognition specificity.

## 5 What Should We Do Next?

A detailed understanding of the strengths and limitations of existing approaches is key for designing the next‐generation of TCR‐epitope recognition prediction tools. Overall, several important observations have emerged from recent studies which should be considered to improve both the accuracy and usability of such tools (Table 3).

**TABLE 3:** Summary of several important observations related to modeling specificity in TCR‐epitope interactions, and the consequences for the design of experiments and computational tools.

Key observations	Implication for the design of experiments	Implications for the design of computational tools	Main relevance
All 1D‐TRMs have failed to generalize to unseen epitopes.	TCRs recognizing as many clinically relevant epitopes as possible should be experimentally identified.	Epitopes can be treated as categories, or equivalently epitope‐specific tools can be used. This significantly reduces the number of parameters that need to be learned.	1D‐TRM
Much of the TCR‐epitope recognition specificity is encoded in V/J usage.	Libraries of TCRs used to screen epitopes should integrate diversity in V/J usage.	Tools need to integrate information about V and J genes and not only focus on CDR3 sequences.	1D‐TRM
TCR‐epitope recognition specificity is encoded in both chains.	TCR‐sequencing of epitope‐specific T cells should always include alpha and beta chains.	Tools need to integrate both chains in their architecture.	1D‐TRM
Little specificity is encoded in the pairing between chains, compared to the specificity encoded in each chain.	Paired TCRαβ remains optimal for training predictors, but unpaired TCRα + TCRβ sequencing of epitope‐specific T cells provides a less expensive and more scalable alternative.	Architectures allowing for training on both paired TCRαβ and unpaired TCRα + TCRβ sequences should be designed.	1D‐TRM
Several TCR repertoire sequencing protocols only provide information on a single chain or on unpaired TCRα + TCRβ.		Despite important intrinsic limitations, prediction tools should ideally be able to score single‐chain TCRs to annotate currently available TCR repertoires from large cohorts.	(1D)/3D‐TRM
TCR repertoires from cohorts of patients will become increasingly large, reaching billions of TCRs.		Prediction tools need to scale to billions of TCR‐epitope pairs.	(1D/)3D‐TRM
The theoretical size of TCR repertoires is many orders of magnitude larger than what can be sequenced in a patient.		Prediction tools may not need to learn all the detailed patterns characterizing epitope‐specific TCRs, as long as they are able to correctly identify the vast majority of non‐binding TCRs.	1D/3D‐TRM
Benchmarking datasets currently use randomly generated negatives.	Experiments should be designed where the non‐binding TCRs are sequenced from the same donor and processed with the same experimental and computational protocols as the epitope‐specific TCRs.	When using TCRs with undetermined specificity as negatives, special care should be taken to avoid biases or batch effects shared between training and test sets. When using swapped negatives, special care should be taken to prevent specificity or clustering patterns shared between training and test sets.	1D‐TRM
Existing databases of TCR‐epitope interactions contain a significant number of false positives.	Experimental methods should be developed to validate reported TCR‐epitope interactions, especially when using these data to benchmark tools.	Computational approaches should be designed to probe the quality of existing data, especially when using these data to benchmark tools. 1D‐TRMs need to tolerate some level of false positives in their training data.	1D/3D‐TRM

*Note:* Parentheses indicate lower relevance, or relevance restricted to a subset of tools.

First, as already mentioned, all 1D‐TRMs have failed to generalize to unseen epitopes [45, 126, 127, 128, 153]. We therefore advocate that efforts should focus on experimentally identifying epitope‐specific TCRs for as many clinically relevant epitopes as possible. On the computational side, this indicates that, as of today, epitopes can be treated as categorical variables in pan‐epitope 1D‐TRMs, or equivalently that epitope‐specific 1D‐TRMs can be used to train specific models for each epitope. This will simplify the architecture and significantly reduce the number of parameters of machine learning predictors by eliminating the need to learn complex and currently poorly understood patterns underlying correlations between epitope and TCR sequences, which would require several orders of magnitude more data [138].

Second, the V and J genes, which fully determine CDR1 and CDR2 residues as well as 80% of CDR3α and 65% of CDR3β residues on average [25], play a central role in epitope recognition specificity [13, 15, 100]. We advocate that 1D‐TRMs should always consider the full TCR information and not focus only on CDR3s. This is especially important for V genes, since many of them have highly similar CDR3 residues (e.g., CASS motif in CDR3β) but show substantial differences in other parts, including CDR1 and CDR2 loops. In terms of experiments, we advocate that libraries of TCRs designed to be selected against epitopes should integrate V/J gene diversity and not only focus on CDR3 sequences. This is especially relevant for synthetic TCR libraries used in phage or yeast display, which up to now have used fixed V and J genes [154, 155].

Third, both the alpha and the beta chains contribute to epitope recognition specificity [15, 57, 131]. We advocate to always sequence alpha and beta chains in epitope‐specific T cells, when using these data for training TCR‐epitope recognition prediction tools, and to include both chains in the architecture of existing 1D‐TRMs.

Fourth, we have recently demonstrated for multiple tools and across hundreds of epitopes that training 1D‐TRMs on actual paired TCRαβ or shuffled TCRαβ pairs preserves their accuracy [156]. Similar observations were made based on the analysis of a set of six epitopes [100]. This does not mean that any combination of alpha and beta chains found in epitope‐specific TCRs will preserve the binding. Instead, it reflects that the likelihood of having a non‐binding TCR with a good alpha and a good beta chain in a realistic repertoire of roughly 10,000 TCRs sequenced from a patient is actually very low. While paired TCRαβ sequences remain the gold standard, we advocate that unpaired TCRα + TCRβ sequencing, which is much cheaper and shows higher sequencing depth, can also be used for generating training data for 1D‐TRM predictors [15, 156].

Fifth, several state‐of‐the‐art 1D‐TRMs and most 3D‐TRMs do not support in their input single‐chain TCRα and/or TCRβ repertoires (Tables 1 and 2). Considering that such data still represent the majority of clinically annotated TCR repertoires, we advocate to include in 1D‐ and 3D‐TRMs tools the possibility to run predictions on single‐chain TCRα or TCRβ repertoires, despite the expected lower accuracy of such predictions.

Sixth, 3D‐TRMs require much heavier computations to build and score 3D models of TCR‐epitope complexes compared to 1D‐TRMs. We advocate that efforts should be made to find ways of accelerating 3D‐TRMs for annotating TCR repertoires from large cohorts of patients reaching billions of TCRs [42] for hundreds of epitopes.

**FIGURE 5:**
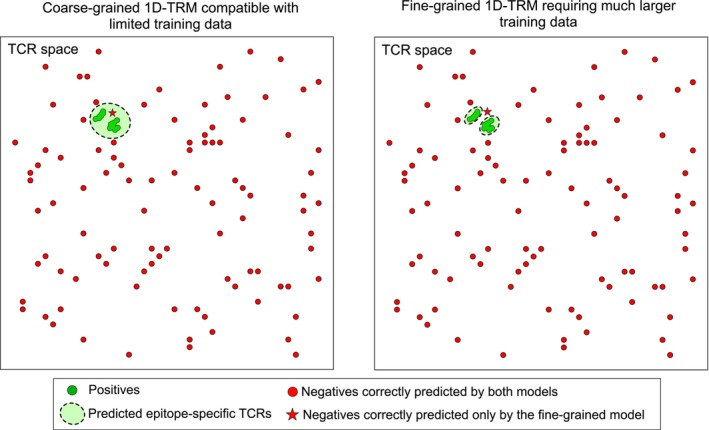
Illustration of 1D‐TRMs with different levels of granularity that require different amount of training data. Both models can correctly identify both the epitope‐specific TCRs and the vast majority of non‐binding TCRs and therefore will result in very similar prediction accuracy when annotating TCR repertoires, even if the coarse‐grained model fails to correctly predict a tiny fraction of the non‐binding TCRs (red cross). We stress that these cartoons aim at illustrating some important properties of the TCR space, but do not recapitulate the high dimensionality of this space.

Seventh, the theoretical diversity of TCR repertoires (at least 10^16^, likely much higher) is many orders of magnitude larger than the size of TCR repertoires that can be sequenced in a patient (10^4^–10^6^). We advocate that complex 1D‐ and 3D‐TRM tools able to capture in theory every single, and possibly quite intricate, pattern characterizing TCRs recognizing a given epitope may not always be needed. In particular, tools that are able to give a good score to most epitope‐specific TCRs and to exclude the vast majority (i.e., 99.9%) of non‐binding TCRs are sufficient for many applications, even if they cannot model some challenging cases of non‐binding TCRs like TCRs with a good alpha and a good beta chain that put together do not preserve the binding [156] (see illustration in Figure 5 for 1D‐TRMs). This is good news for the field, since accurately learning all possibly very complex patterns characterizing epitope‐specific TCRs with 1D‐TRMs requires hundreds if not thousands of epitope‐specific TCRs, which are currently not available for most epitopes.

Eighth, most existing benchmark datasets use randomly generated negatives and prevalently swapped negatives. We advocate that efforts should focus on generating negatives that reflect realistic applications of TCR‐epitope predictions. One practical way to generate such data is to use peptide–MHC tetramers and sequence both the tetramer+ and tetramer− T cells from the same biological sample. In this way, issues related to batch effects or other biases between positives and negatives will be minimized and the structure of the negatives will not differ from the expected structure of baseline TCR repertoires.

Finally, existing databases of TCR‐epitope interactions have been reported to contain a substantial proportion of false positives [15, 25, 48, 106]. While some false positives are likely impossible to avoid, experimental and computational approaches should be developed to carefully validate these data, especially when using them to benchmark 1D‐ and 3D‐TRMs. These include scalable methods to validate TCR‐epitope interactions [106], or computational methods that can pinpoint issues in TCR sequences such as wrong V/J gene annotations [25], or detect unexpected lack of specificity patterns in TCRs reported to interact with a given epitope in some studies [15, 48].

## 6 Conclusion

Modeling TCR‐epitope recognition specificity has been seen as a major challenge in immunology and cancer immunotherapy. Since the first studies demonstrating that clear patterns exist in epitope‐specific TCRs and can be used to distinguish them from baseline/background TCR repertoires [13, 43], several key developments have been made. Many machine learning or distance‐based algorithms have been designed and several of them show good predictive power for epitopes with abundant and reliable training data. In parallel, major improvements in predicting TCR‐epitope 3D structures with tools like AlphaFold can be used to accurately predict TCR‐epitope interactions in several, though not all, cases. To further improve such predictions, we have outlined some key observations. First, a major aim for 1D‐TRMs should be to generate reliable training data for as many clinically relevant epitopes as possible. Second, unless a massive increase in the coverage of the epitope space is reached, attempts to learn correlations between TCR and epitope sequences and extrapolate 1D‐TRMs predictions to unseen epitopes should be carefully assessed in terms of feasibility. Third, all experimental and computational approaches should use information from both TCR chains, include V and J gene information and not focus only on CDR3 sequences, and provide MHC information and not focus only on peptide sequences. Fourth, improving the computational efficiency of 3D‐TRMs will be important to annotate large repertoires comprising billions of TCRs from cohorts of patients with hundreds of different epitopes. Finally, enhancing the quality of the data used to train 1D‐TRMs and to benchmark 1D‐ and 3D‐TRMs will be critical to further improve existing approaches and better understand when predictions can be trusted and when they fail.

## Funding

The work is supported by the SNF Project Grant (320030‐231333) and the ISREC foundation.

## Conflicts of Interest

The authors declare no conflicts of interest.

## Supporting information


**Table S1:** List of existing 1D‐TRMs with a publicly available command‐line implementation or a web interface as well as some minimal documentation. Parentheses indicate optional features. In the last column, “P” stands for paired TCRab sequences, “A” stands TCRa sequences and “B” stands for TCRb sequences.

**Table S2:** List of existing 3D‐TRMs with a publicly available command‐line implementation or web interface as well as some minimal documentation. CNN, Convolutional neural network; GNN, Graph Neural Network. In the last column, “P” stands for paired TCRab sequences and “B” stands for TCRb sequences.

## Data Availability

The data that supports the findings of this study are available in Tables S1 and S2.
